# Disordered Eating and Exercise Addiction Among Former Student-Athletes: Contribution of Athletic Experience and Personality

**DOI:** 10.3390/nu18132054

**Published:** 2026-06-24

**Authors:** Juliette Maurin, Véronique Boudreault, Olivier Laverdière

**Affiliations:** 1 Department of Psychology, Faculty of Humanities, Université de Sherbrooke, Sherbrooke, QC J1K 2R1, Canada; 2 Faculty of Human Kinetics, Université de Sherbrooke, Sherbrooke, QC J1K 2R1, Canada; veronique.boudreault2@usherbrooke.ca

**Keywords:** sport retirement, retired athletes, eating disorder symptoms, compulsive exercise, perfectionism

## Abstract

**Background/Objectives**: Sport retirement entails many adjustments for varsity student-athletes, including changes in identity and body-related experiences, potentially increasing their vulnerability to disordered eating (DE) and exercise addiction. This study aimed to (1) compare the severity of DE and exercise addiction symptoms between former varsity student-athletes and former non-athlete students, and (2) examine whether the associations between personality traits and these symptoms differ across groups. **Methods**: A total of 88 former varsity student-athletes and 69 former non-athlete students completed an online questionnaire between January and September 2025. **Results**: Former student-athletes reported more symptoms of exercise addiction (*p* = 0.025), a tendency to report lower DE associated with drive for muscularity (*p* = 0.074), and similar levels of DE associated with drive for thinness and symptoms of orthorexia (*p* = 0.273 and *p* = 0.376, respectively) compared to the control group. Furthermore, perfectionism was significantly associated with all dependent variables. Moderation analyses revealed significant interactions between perfectionism and group (*p* = 0.048 for drive for thinness and *p* = 0.044 for drive for muscularity), indicating that the association between perfectionism and DE associated with drive for thinness and drive for muscularity is significant in the control group but not in former student-athletes. **Conclusions**: These findings underscore the need to prevent and detect symptoms of exercise addiction as well as different forms of DE throughout an athletic career and during retirement. Interventions targeting perfectionism, such as fostering acceptance of body-related experiences, clarifying personal values, and developing greater body awareness, may help support student-athletes and reduce vulnerability to exercise addiction and DE.

## 1. Introduction

### 1.1. Disordered Eating

The main eating disorders (EDs) defined in the DSM-5 are anorexia nervosa and bulimia nervosa [1]. Both of these widely studied EDs are characterized by an excessive and intrusive pursuit of thinness [2,3]. Orthorexia and muscle dysmorphia are not yet included in the DSM-5 [1] but are common subclinical presentations that are becoming increasingly well defined in the literature. Orthorexia consists of an intrusive preoccupation with healthy eating, leading to the avoidance of any food that fails to meet the person’s criteria [4]. Muscle dysmorphia (also called bigorexia, reverse anorexia, or Adonis complex; [5]) refers to the pathological impression that the body is not sufficiently lean and muscular [6]. To confirm these diagnoses, people who present symptoms must meet a set of specific symptomatic and severity criteria, which excludes those who experience related symptoms without fully meeting the threshold for a clinical diagnosis [7]. It is therefore imperative to consider not only the clinical forms of EDs, but also the subclinical forms, as it may progress to clinical forms if left untreated. Moreover, although research has long focused on DE associated with drive for thinness, Fairburn’s transdiagnostic model suggests that different forms of DE share several common underlying mechanisms, including the overvaluation of weight, body shape, or the ability to control these elements [8]. While their behavioral manifestations may differ (e.g., DE associated with drive for thinness, DE associated with drive for muscularity, symptoms of orthorexia), these manifestations converge around a dysfunctional relationship with the body and eating behaviors. Thus, considering multiple forms of DE allows for a more comprehensive and nuanced understanding of DE as a multidimensional construct [9,10,11]. In this study, the term “disordered eating” (DE) encompasses both EDs and subclinical forms, and includes DE associated with drive for thinness (the clinical forms of which are generally associated with diagnoses of anorexia or bulimia), DE associated with drive for muscularity (the clinical form of which may be associated with muscle dysmorphia), and symptoms of orthorexia. These issues represent behaviors aimed at controlling weight and body composition, in which physical exercise can become a central lever. Thus, DE, particularly that associated with drive for thinness, has been widely associated with symptoms of exercise addiction [12,13].

### 1.2. Exercise Addiction

Exercise addiction is defined as “a dysfunctional behavior characterized by exaggerated training, loss of control over exercise behaviour, and negative life consequences that could be physical, psychological or social, or a combination of the three” [14,15,16] (p. 1). Kern conceptualizes problematic physical activity on a continuum ranging from “excessive practice” to “addictive practice”, with dependence and compulsion positioned between these two extremes [17] (see Kern (2021) [17] for a visual representation of the continuum of problematic physical activity). In the present study, the term exercise addiction refers to the most severe end (i.e., addictive practice), encompassing both compulsive and dependence dimensions, while “symptoms of exercise addiction” capture the full range of Kern’s continuum. Several core features have been identified to further characterize exercise addiction, including tolerance; withdrawal symptoms; continuing to exercise despite knowing that it causes physical, psychological, or social problems; inability to reduce physical exercise; significant time spent preparing for, performing, and recovering from exercise; and elimination of other activities in order to devote more time to exercise [12,13]. Although the definition of exercise addiction is relatively well established [16], conceptual ambiguity remains regarding its primary and secondary forms, particularly concerning the relevance and clinical utility of distinguishing between them [17].

Exercise addiction has traditionally been divided into primary and secondary forms based on its association with DE [16]. Primary exercise addiction refers to excessive and compulsive exercise that occurs independently of another disorder. In contrast, secondary exercise addiction refers to cases in which exercise addiction is closely linked to DE, functioning as a strategy to control body weight and shape [18]. Given the strong association between exercise addiction and DE [12,13] as well as the limited understanding of individuals presenting with primary exercise addiction, some authors have questioned whether primary exercise addiction truly exists as a distinct condition [17,19]. However, other studies have reported prevalence rates of primary exercise addiction, thereby supporting that both forms exist [12,18,20]. Regardless of this distinction, exercise addiction is associated with adverse consequences for health and well-being, including injuries, psychological distress, and impaired functioning [17]. This supports the relevance of examining exercise addiction as a broader construct that may emerge in different contexts (in a DE context or not) [17].

Despite this lack of consensus regarding exercise addiction, studies have identified risk factors specific to certain populations, particularly current and former athletes [21,22].

### 1.3. Transition out of Varsity Sport: Risk Factors for DE and Exercise Addiction

During a sport career, certain types of sports may be more conducive to the development of DE and exercise addiction symptoms [13,23,24]. For instance, athletes in high-energy sports, such as endurance sports, may be at greater risk of developing exercise addiction symptoms [13,23]. Athletes in esthetic, endurance, and weight-class sports may be more likely to develop DE due to the specific physical demands of these types of sports (e.g., results based on physical esthetics in esthetic sports) [24]. During sport retirement, defined as “the process of transition from participation in competitive sport to another activity or set of activities” [25] (p. 1), the internalization of body-related pressures from the sport environment may combine with societal body-related pressures, which become increasingly salient in the post-sport context. As a result, athletes may experience a “paradoxical transition” [10]. Furthermore, if athletic body ideals are similar to societal body ideals, the risk of developing DE would be increased by distancing the athlete not only from their athletic body ideal, but also from the societal body ideal [10,13]. Some studies also suggest that difficulties related to body image can arise in former athletes even without actual weight gain [10,26]. For example, Papathomas et al. found that more than half of the former female athletes participating in esthetic sports reported being dissatisfied with their weight and trying to lose weight, even though 75% of the athletes were considered to be at a “healthy” weight [26]. Thus, participation in certain sport contexts that emphasize a specific athletic body ideal may contribute to the persistent internalization of body-related pressures, even after the end of an athletic career. This internalization may, in turn, manifest in different forms of DE [8].

Furthermore, during the transition to retirement, varsity student-athletes not only leave their academic environment but also lose their athletic and student statuses that have defined them for several years [27]. Indeed, 98% of varsity student-athletes retire from sports after graduating from university [28]. As a result, student-athletes who retire from sports may experience significant changes not only in their bodies, but also in their identities, which can have a beneficial or detrimental effect on their body image and lifestyle habits [10,26]. Given these profound identity changes, some athletes may attempt to preserve their athletic identity by maintaining behaviors and attitudes closely tied to their former athletic lifestyle [10,13]. This tendency to maintain sport-related habits (e.g., structured eating, frequent and intense training, high energy expenditure, pursuit of an athletic body) after retirement may increase former student-athletes’ vulnerability to DE and exercise addiction symptoms [10,13]. These difficulties may also reflect a continuation of behaviors acquired during their athletic career [22,29]. For example, the qualitative study by Plateau et al. shows how athletes can relearn healthy eating habits and feel liberated in their diet during their retirement from sport. These results suggest that other athletes may, on the contrary, continue to follow restrictive diets learned during their sporting careers [29].

Although few studies on DE and exercise addiction exist among former athletes, retirement from sport is recognized as a period conducive to the maintenance or development of DE and exercise addiction symptoms [10,13]. Another factor that can influence the development of DE and exercise addiction is athletes’ personality [16].

### 1.4. Personality

Studies have identified personality traits associated with DE and exercise addiction. To better understand their development, facilitate diagnosis, and tailor treatments (e.g., [30,31]), several authors use the five-factor model (“Big Five”) [31,32,33]. For example, Ali et al. found that traits associated with openness could be a risk factor for DE in student-athletes, while for non-athlete students, traits associated with conscientiousness could be a risk factor and level of extraversion a protective factor, suggesting an interaction between personality and experience as an athlete [34]. Regarding exercise addiction, most studies have shown that exercise addiction is positively associated with neurotic traits [22,33,35]. Although the five-factor personality model is a universal model, perfectionism, which has been studied extensively in the context of DE, is only partially assessed by this model [36].

In the general population, and particularly among athletes, perfectionism has been widely associated with DE and exercise addiction [12,24,35,37]. Perfectionism is a multidimensional personality trait characterized by excessively high standards, a tendency to engage in critical evaluations of oneself and others, and a fear of mistakes or negative evaluations [38,39]. Hewitt and Flett conceptualized perfectionism as comprising three dimensions: self-oriented perfectionism (SOP), other-oriented perfectionism (OOP), and socially prescribed perfectionism (SPP) [39]. SOP is characterized by perfectionistic behaviors directed toward oneself (e.g., setting high personal standards); OOP involves expectations toward others (e.g., setting unrealistic standards for others), and SPP is characterized by the need to meet expectations imposed by others [39]. Only SOP was considered in the present study given its association with DE and exercise addiction [36,40]. SOP was also selected because it captures internally directed perfectionistic standards that may persist beyond the athletic career and contribute to the maintenance of rigid exercise, eating, or body-related behaviors after sport retirement.

Although personality traits have already been looked at in several studies on DE and exercise addiction, authors raise the need to better understand the personality factors associated with exercise addiction [16,17]. Furthermore, a better understanding of the personality factors associated with exercise addiction and those associated with DE could shed light on the dynamics linking these two issues [12].

### 1.5. Objectives

Although DE and exercise addiction are receiving increasing attention, few studies have examined these issues among former student-athletes, despite their unique circumstances that may heighten specific risks [10,13]. Furthermore, it is important not only to investigate how personality, in interaction with athletic experience, contributes to DE and exercise addiction, but also to explore the underlying personality factors that may link these two conditions [16,17,41]. This study therefore aims to better understand the contribution of athletic experience and personality traits to DE and exercise addiction symptoms in former student-athletes. Specifically, this study has two main objectives: (1) to compare the severity of DE and exercise addiction symptoms between former varsity student-athletes and former non-athlete students, and (2) to examine whether the associations between personality traits and these symptoms differ across groups. Former student-athletes are expected to exhibit greater severity of DE and exercise addiction symptoms. It is also expected that perfectionism will be associated with DE, and that neurotic traits and perfectionism will be associated with exercise addiction. Finally, we posit that certain personality traits (particularly perfectionism) are more strongly associated with the variables studied in former student-athletes than in control group participants.

## 2. Materials and Methods

### 2.1. Participants and Procedure

This research, approved by the Ethics Committee for Research in the Humanities and Social Sciences at Université de Sherbrooke (#2024-4630), is cross-sectional in nature. Two groups of participants were investigated: A group of former varsity student-athletes and a control group composed of former non-athlete students. To participate, former student-athletes had to: (a) have been a university student while participating in competitions in the Réseau du Sport Étudiant du Québec (RSEQ) circuit or having been identified as an “excellence”, “élite” or “relève” athlete by a Québec sports federation, (b) have stopped participating in competitive sports at the level achieved during their athletic career within the last 10 years, and (c) have left university within the last 10 years. For the control group, participants had to have left university within the last 10 years and not have competed in the RSEQ circuit or been identified as an “excellence”, “élite” or “relève” athlete by a Québec sports federation.

Recruitment took place from January to September 2025, aided by various organizations with strong connections to active and former student-athletes. The project was also published on social media. Participants completed an online questionnaire lasting approximately 25 min on a secure LimeSurvey online platform. All participants were presented with an informed consent form at the start of the online questionnaire, and consent was obtained prior to their participation. A total of 562 people accessed the survey: 363 participants were removed because they did not complete the main variables (304 who simply clicked on the questionnaire link without completing any questions, and 59 participants who completed a few sociodemographic questions); 41 participants were removed because they did not meet the inclusion criteria; and 1 participant did not give their consent to participate. The final sample therefore included 157 participants: 88 former varsity student-athletes and 69 former non-athlete students. Comparisons between excluded participants who completed the primary sociodemographic items (*n* = 59) and retained participants revealed no significant differences in age, sex, sexual orientation, or educational level.

### 2.2. Instruments

Participants answered a series of sociodemographic questions about their age, sex assigned at birth, type of sport, years of participation in primary sport, highest level of competition, years since retirement and type of sport retirement.

Eating Disorder Examination Questionnaire

The French version of the Eating Disorder Examination Questionnaire (EDE-Q) is a 22-item tool used to assess the presence of DE associated with drive for thinness in the general population [42,43]. A seven-point scale ranging from 0 (never or not at all) to 6 (every day or strongly) allows participants to record the frequency and intensity with which they have experienced DE associated with drive for thinness over the past four weeks. The total score is the average of the scores obtained on the subscales. A total score greater than 2.5 was considered an indicator of ED, based on recent studies conducted among athletic populations and Canadian populations [44,45]. In the present study, the overall score was analyzed as a continuous variable, but the dichotomous score was used to better describe the sample. The EDE-Q demonstrates good internal consistency (Cronbach’s alpha between 0.89 and 0.90) according to a study conducted with university students [46]. Among athletes, Lichtenstein et al. report good construct validity of the instrument, including high convergent validity (*r* = 0.729) [20]. In this study, McDonald’s omega for the overall score was 0.95.

Eating for Muscularity Scale

The French version of the Eating for Muscularity Scale (EMS) assesses the presence of DE associated with drive for muscularity [47]. This tool contains 27 items, answered on a scale ranging from 0 (never) to 6 (every day). The total score is obtained by averaging all items. In the present study, the overall score was analyzed as a continuous variable, with higher scores indicating greater severity of DE. The original version was validated in the general population and demonstrated good internal consistency (α = 0.95) as well as moderate to strong correlations (*r* = 0.43 to *r* = 0.71) with related measures (e.g., the Muscularity Dissatisfaction Subscale of the Male Body Attitudes Scale [48]), suggesting strong convergent validity [47]. The French version was developed using the forward–backward translation method. The forward translation was conducted by two bilingual individuals with expertise in training and nutrition. The backward translation was carried out by an English language teacher, a kinesiologist, and a third bilingual expert in the fields of training and DE. Although this version has not yet undergone full psychometric validation in a French-speaking population, it demonstrated excellent preliminary internal consistency in the present sample, with a McDonald’s omega of 0.92 for the overall score.

French Orthorexia Scale

To measure orthorexia symptoms, the French Orthorexia Scale (Échelle Française d’orthorexie, EFO-12) was used [49]. This questionnaire, specifically developed for the French-speaking cultural context, was designed to address the limitations of existing instruments used to measure orthorexia symptoms, and its development was based on the most recent diagnostic criteria for orthorexia [49]. It includes 12 items with a four-point scale ranging from 0 (does not apply to me at all) to 3 (applies to me completely). The total score is obtained by adding up the scores for all items, resulting in a continuous score ranging from 0 to 36, where a higher score indicates greater difficulties. The EFO-12 demonstrates good psychometric properties, including good internal consistency (α = 0.82) and good convergent validity (*r* = 0.57) [49]. McDonald’s omega was 0.83 for this study.

Exercise Dependence Scale-Revised

Participants’ level of exercise addiction was measured by the French version of the Exercise Dependence Scale-Revised (EDS-R) [50,51]. This 21-item questionnaire uses a six-point scale ranging from 0 (never) to 5 (always), with higher scores on the EDS-R indicating higher levels of exercise addiction. Scores of 5 or 6 are considered indicative of addiction, scores of 3 or 4 are considered indicative of symptoms, and scores of 1 or 2 are considered indicative of no symptoms. The total score is obtained by adding up the scores for all items on the questionnaire. This scale also provides a categorical score that distinguishes between individuals at risk of exercise addiction, those who exhibit symptoms, and those who exhibit no symptoms. In this study, the categorical score was used to determine the prevalence of probable primary and secondary exercise addiction. Participants presenting symptoms of exercise addiction (“symptomatic”) without risk of DE were classified as probable primary exercise addiction cases, whereas those presenting symptoms of exercise addiction with risk of DE were classified as probable secondary exercise addiction cases. The overall EDS-R score was analyzed as a continuous variable for main analyses. According to Kern’s study, the different dimensions of this instrument demonstrate satisfactory internal consistency (Cronbach’s alphas ranging from 0.60 to 0.90), and the instrument shows convergent validity between 0.69 and 0.75 [51]. McDonald’s omega for the total score was 0.94 in this study.

Self-Oriented Perfectionism

The French short version of the SOP dimension (five items) of the Multidimensional Perfectionism Scale (MPS) was used to assess participants’ level of perfectionism [39,52]. Participants rated the items on a Likert scale ranging from 0 (strongly disagree) to 6 (strongly agree). The SOP score is obtained by adding the scores of the items on the subscale. Higher scores indicate greater intensity of SOP traits. The SOP subscale of the MPS demonstrates good convergent validity with the Attitude Toward Self Scale (*r* = 0.72), and a good internal consistency (Cronbach’s alpha of 0.86) [39,53]. In the present study, the SOP dimension had a McDonald’s omega of 0.99. Although this coefficient indicates excellent internal consistency, it may also reflect substantial overlap among items.

Big Five Inventory

The French version of the Big Five Inventory (BFI) was used to measure participants’ personality traits [54,55]. This instrument consists of 44 items divided into five dimensions: Extraversion (E), Agreeableness (A), Conscientiousness (C), Neuroticism (N), and Openness (O). Participants rate each item on the BFI on a 5-point scale ranging from 1 (strongly disagree) to 5 (strongly agree). The score for each dimension is obtained by averaging the items in that dimension. Higher scores in certain dimensions indicate that the participant exhibits more traits associated with those dimensions. In a validation study conducted with a French student population, this instrument shows acceptable to good internal consistency across its dimensions (Cronbach’s alphas ranging from 0.74 to 0.82) and convergent validity of 0.74 with the NEO-PI-R [55]. In the present study, McDonald’s omegas were 0.87 (E), 0.71 (A), 0.87 (C and N), and 0.80 (O).

### 2.3. Analyses

#### 2.3.1. Preliminary Analyses

Power analyses were performed using G*Power software version 3.1 to determine the number of participants required to perform a multivariate analysis of covariance (MANCOVA) and multiple linear regression [56]. These analyses suggested that for a *p*-value of 0.05, a power of 0.80 and a small effect size, a sample of 126 participants was needed to detect a significant effect in a two-group MANCOVA with four dependent variables. Similarly, for a *p*-value of 0.05, a power of 0.80 and a medium effect size, a sample of 114 participants was needed to detect a significant effect in a multiple linear regression with nine predictors.

All analyses were performed using SPSS software version 29.0 and the PROCESS extension version 4.2 [57,58]. Missing data were handled using listwise deletion, such that only participants with complete data on all variables included in each analysis were retained. Consequently, sample sizes varied slightly across analyses. Descriptive analyses (e.g., frequencies, percentages, means, standard deviations, correlations, chi-square tests, *t*-tests) were performed to describe the sample (see Table 1, Table 2 and Table A1). Most variables appeared to be normally distributed, except for the variable of DE associated with drive for muscularity (asymmetry = 2.775; skewness = 10.050). Thus, in order to reduce the influence of extreme values for the main analyses, this last variable was winsorized at the 95th percentile. Sensitivity analyses conducted using the non-winsorized variable yielded the same conclusions. However, the non-winsorized distribution showed violations of regression assumptions due to extreme values; therefore, the winsorized variable was retained for primary analyses.

#### 2.3.2. Main Analyses

In order to compare groups of participants on DE and exercise addiction symptoms (objective 1), a MANCOVA and a discriminant function analysis were performed. One independent variable (the group) and four dependent variables (DE associated with drive for thinness, DE associated with drive for muscularity, symptoms of orthorexia, and symptoms of exercise addiction) were included in the analyses with sex as a covariate. The procedure proposed by Smith et al. was followed to ensure compliance with the MANCOVA assumptions [59]. The Box test was used to verify the equality of the covariance matrices (*p* = 0.175).

To examine the contribution of personality to DE and exercise addiction symptoms across the two groups, four regression analyses were conducted with sex included as a covariate and the five personality factors, SOP, and group entered as predictors. Subsequently, a moderation analysis was performed for DE and exercise addiction symptoms to assess the interaction between group and the predictor that emerged as significant in the regression. These moderation analyses included the same predictors as for the regressions, plus the interaction factor. The assumptions of these analyses were met (no excessive collinearity between predictors, normality of residuals, no outliers). However, the scatterplot of standardized residuals seemed to indicate heteroscedasticity. Regression and moderation analyses were conducted in PROCESS using the Davidson–MacKinnon option to estimate robust standard errors [58]. All continuous variables were centered for moderation analyses.

## 3. Results

### 3.1. Sample Characteristics

Participants’ sociodemographic characteristics can be found in Table 1 for both groups of participants, while Table 2 presents descriptive statistics of the ten variables of interest. Between-group comparisons (chi-square tests and *t*-tests) indicated no significant differences between the two groups on all sociodemographic variables and most study variables. However, former student-athletes exhibited significantly higher levels of SOP and lower levels of neuroticism compared to the control group.

**Table 1:** Participants’ Characteristics.

Variable		Former Student-Athletes	Former Non-Athlete Students	Total
Age (*n*(A) = 87 *n*(C) = 65)	Mean SD Min–Max	28.8 3.54 22–39	29.3 3.23 22–36	29.0 3.41 22–39
Sex (*n*(A) = 88 *n*(C) = 69)	Women Men	63 (71.6%) 25 (28.4%)	57 (82.6%) 12 (17.4%)	120 (76.4%) 37 (23.6%)
Type of sport (*n*(A) = 86)	Team Endurance Esthetic Weight Categories Technical	36 (41.9%) 34 (39.5%) 7 (8.1%) 1 (1.2%) 8 (9.3%)	-	-
Years of participation in the primary sport (*n*(A) = 87)	Mean SD Min–Max	11.94 5.11 1–25	-	-
Highest level of competition (*n*(A) = 88)	Regional Provincial National North American circuit International	2 (2.3%) 22 (25.0%) 46 (52.3%) 3 (3.4%) 15 (17.0%)	-	-
Years since retirement (*n*(A) = 88)	Mean SD Min–Max	4.43 3.02 0–10	-	-
Type of retirement (*n*(A) = 88)	Normative Non-normative	54 (61.4%) 34 (38.6%)	-	-
Risk of ED (*n*(A) = 88 *n*(C) = 69)	Yes No	12 (13.6%) 76 (86.4%)	14 (20.3%) 55 (79.7%)	26 (16.6%) 131 (83.4%)
Exercise Addiction (*n*(A) = 86 *n*(C) = 68)	Addicts Symptomatic Non-symptomatic Primary Secondary	0 17 (19.8%) 69 (80.2%) 13 (15.1%) 4 (4.7%)	0 9 (13.2%) 59 (86.8%) 5 (7.4%) 4 (5.9%)	0 26 (16.9%) 128 (83.1%) 18 (11.7%) 8 (5.2%)

Note. *n*(A) = number of former student-athletes; *n*(C) = number of participants form the control group.

**Table 2:** Descriptive Statistics of the Main Variables for Each Group.

Variable	*N*	*M*	*SD*	Min–Max	Skewness; Kurtosis
EDE-Q (A)	88	1.274	1.071	0.00–4.84	1.178; 1.105
EDE-Q (C)	69	1.513	1.353	0.00–5.33	1.199; 0.755
EMS (A)	88	0.398	0.380	0.00–1.63	1.398; 1.593
EMS (C)	69	0.528	0.530	0.00–1.63	0.986; −0.251
EFO-12 (A)	88	8.398	4.956	1.00–27.00	1.039; 1.212
EFO-12 (C)	69	7.580	5.666	0.00–25.00	1.127; 0.981
EDS-R (A)	88	27.330 *	15.129	2.00–69.00	0.726; 0.017
EDS-R (C)	69	20.942 *	17.445	0.00–77.00	1.054; 0.757
SOP (A)	87	19.678 *	5.888	2.00–29.00	−0.965; 0.941
SOP (C)	67	16.328 *	8.140	0.00–30.00	−0.066; −0.928
BFI_E (A)	85	3.303	0.924	1.38–5.00	−0.170; −0.668
BFI_E (C)	65	3.256	0.857	1.75–5.00	0.342; −0.828
BFI_N (A)	85	2.737 *	0.841	1.00–4.25	−0.340; −0.672
BFI_N (C)	65	3.226 *	0.834	1.50–4.75	−0.282; −0.658
BFI_A (A)	85	3.915	0.498	2.10–4.80	−0.687; 1.074
BFI_A (C)	65	3.888	0.561	2.20–4.90	−0.528; 0.393
BFI_C (A)	85	3.983	0.636	2.00–5.00	−0.702; 0.604
BFI_C (C)	65	3.761	0.812	1.78–5.00	−0.581; 0.004
BFI_O (A)	85	3.476	0.662	1.78–5.00	−0.278; 0.243
BFI_O (C)	65	3.675	0.629	2.10–4.90	−0.335; −0.020

Note. (A) Former student-athletes; (C) Control group. EDE-Q = Eating Disorder Examination Questionnaire; EMS = Eating for Muscularity Scale; EFO-12 = Échelle Française d’Orthorexie; EDS-R = Exercise Dependence Scale Revised; SOP = Self-Oriented Perfectionism; BFI_E = Extraversion dimension of BFI; BFI_N = Neuroticism dimension of BFI; BFI_A = Agreeableness dimension of BFI; BFI_C = Conscientiousness dimension of BFI; BFI_O = Openness dimension of BFI. * Significant difference between groups at *p* < 0.05.

### 3.2. Group Differences

The results of the MANCOVA, conducted on all 157 participants, revealed a significant difference between the two groups of participants (former student-athletes and controls) across all dependent variables (Wilks’ *Λ* = 0.90, *F*(4, 151) = 4.33, *p* = 0.002, *ηp*^2^ = 0.103) but no difference between sex (Wilks’ *Λ* = 0.98, *F*(4, 151) = 0.95, *p* = 0.438, *ηp*^2^ = 0.024). Subsequently, the discriminant function analysis was significant (Wilks’ *Λ* = 0.89 *χ*^2^(4) = 17.74, *p* = 0.001), indicating an overall multivariate difference between the groups. The highest correlations with the discriminant function came from exercise addiction symptoms (*r* = 0.56) and DE associated with drive for muscularity (*r* = −0.41), followed by DE associated with drive for thinness (*r* = −0.28) and symptoms of orthorexia (*r* = 0.22). Former student-athletes had significantly higher exercise addiction scores than the control group (*p* = 0.025). DE associated with drive for muscularity was the second strongest contributor to group differentiation, with a trend toward higher scores in the control group (*p* = 0.074). In contrast, the groups did not differ significantly in DE associated with drive for thinness or symptoms of orthorexia (*p* = 0.273 and *p* = 0.376, respectively). The function correctly classified 67.5% of cases, exceeding the proportional chance criterion based on group distribution (56%).

### 3.3. Contribution of Personality

**Figure 1:**
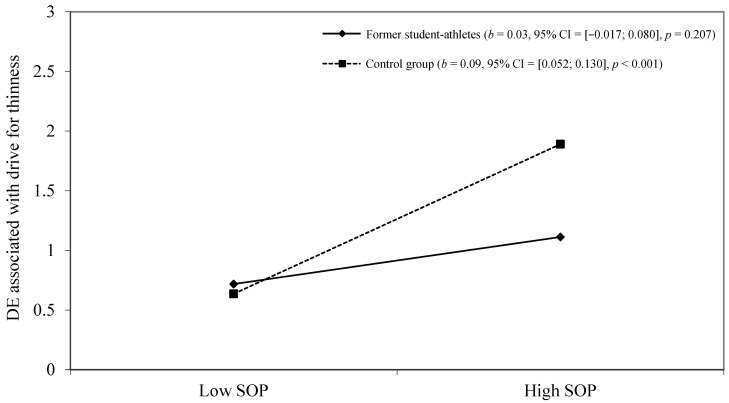
Moderating effect of the group on the association between SOP and DE associated with drive for thinness.

**Figure 2:**
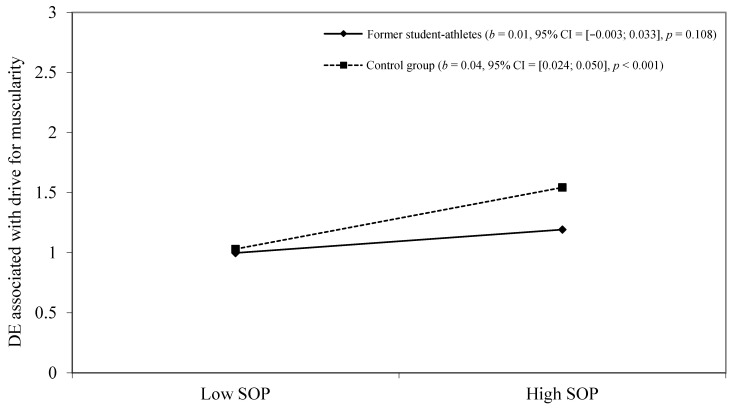
Moderating effect of the group on the association between SOP and DE associated with drive for muscularity.

Regarding DE associated with drive for thinness and with drive for muscularity, the linear regression models were significant (*p* < 0.001 and *p* = 0.002, respectively). Specifically, only SOP was significantly associated with DE associated with drive for thinness and DE associated with drive for muscularity. Both moderation models were also significant (*p* < 0.001 for both models), and the interaction between SOP and group was significant in the models predicting DE associated with drive for thinness and DE associated with drive for muscularity (*p* = 0.048 and *p* = 0.044, respectively), accounting for 2.8% of the variance in these outcomes in both models. The simple effects analysis revealed that SOP was positively correlated with DE associated with drive for thinness and drive for muscularity in the control group (*b* = 0.091, *p* < 0.001, IC95% [0.052; 0.130]; *b* = 0.037, *p* < 0.001, IC95% [0.024; 0.050], respectively), but not in the group of former student-athletes (*b* = 0.031, *p* = 0.207, [−0.017; 0.080]; *b* = 0.015, *p* = 0.108, [−0.003; 0.033], respectively). These results suggest that the effect of perfectionism on DE associated with drive for thinness and with drive for muscularity differs between the two groups. Furthermore, the moderation model concerning DE associated with drive for muscularity revealed that even when sex, personality factors and the interaction between group and perfectionism were taken into account, group remained a significant predictor of DE associated with drive for muscularity. The regression and moderation models are reported in Table 3 and Table 4, and the interaction effects are shown in Figure 1 and Figure 2.

Finally, multiple linear regression models for orthorexia symptoms and exercise addiction symptoms were significant (*p* < 0.001 for both models). In both models, only SOP was significantly associated with orthorexia symptoms and exercise addiction symptoms. In the moderation models, the interaction between SOP and group was non-significant for orthorexia symptoms (*p* = 0.144) and exercise addiction symptoms (*p* = 0.651), adding nothing to the initial regression models. Thus, higher levels of SOP were associated with more symptoms of orthorexia and of exercise addiction, controlling for sex and other personality factors and independent of experience as a varsity student-athlete. The regression models are reported in Table 5 and Table 6.

## 4. Discussion

The main objective of this study was to better understand the contribution of athletic experience and personality traits to DE and symptoms of exercise addiction in former student-athletes. The first specific objective was to compare the severity of DE and exercise addiction symptoms between former varsity student-athletes and former non-athlete students. Overall, former student-athletes reported higher exercise addiction symptoms, whereas they tended to report lower DE associated with drive for muscularity; this pattern partially supported the initial hypothesis. Although few studies on former student-athletes have included a control group, the higher level of exercise addiction symptoms is consistent with work emphasizing the distinct context of former athletes and their population-specific risk factors [13,60]. In contrast, the lower DE associated with drive for muscularity diverges from the limited evidence suggesting that athletes may be particularly vulnerable to this form of DE [37]. The second specific objective was to compare the contribution of personality to DE and exercise addiction symptoms across the two groups. For all the variables studied, perfectionism emerged as a significant predictor, supporting the initial hypothesis and aligning with prior findings [12,37,61]. The sections that follow interpret these results separately for each outcome (DE and exercise addiction) and then consider potential mechanisms that may account for similarities and differences between groups.

### 4.1. Exercise Addiction Symptoms

Former varsity student-athletes showed increased symptoms of exercise addiction compared to the control group. However, this difference was no longer significant after accounting for perfectionism. A potential explanatory hypothesis is that perfectionism may contribute to the association between former varsity sport participation and exercise addiction symptoms, rather than athletic experience alone explaining this vulnerability. SOP, characterized by maintaining high standards for oneself, is associated with strong athletic commitment [62,63]. Accordingly, a high level of perfectionism may have propelled former student-athletes toward varsity sports. This hypothesis is supported by the significantly higher level of perfectionism among former student-athletes compared to the control group. However, since perfectionism was significantly associated with exercise addiction symptoms, former varsity student-athletes may have exhibited more symptoms of exercise addiction given their higher level of perfectionism. Although recent studies have suggested associations between perfectionism, sport commitment, and exercise addiction among athletes [64], this hypothesis should be tested in longitudinal studies examining the indirect effect of student-athlete status on exercise addiction symptoms through perfectionism. These findings are nonetheless consistent with the literature documenting a strong association between perfectionism and exercise addiction [30,35,64], and shed new light on the high prevalence of exercise addiction in samples of athletes [21].

### 4.2. Disordered Eating Associated with Drive for Muscularity and Drive for Thinness

Contrary to the initial hypothesis, former student-athletes tended to report lower DE associated with the drive for muscularity than controls, while the two groups did not differ on DE associated with drive for thinness. This pattern suggests that the two forms of DE may reflect different normative pressures across contexts rather than a single, uniform vulnerability to DE. One plausible hypothesis is that former student-athletes were primarily socialized in a performance-oriented environment, where body practices are evaluated through their perceived impact on athletic output [65]. In that context, behaviors typically aligned with drive for thinness can be framed as functional or performance-relevant (e.g., weight reduction or leanness as “optimization”), making them more easily normalized within training settings. By contrast, behaviors more characteristic of drive for muscularity, particularly those implying large fluctuations in mass or deliberate cycles of “bulking” and “cutting”, may be viewed as less compatible with endurance or esthetic performance demands, and therefore less reinforced in those sport cultures. This hypothesis is consistent with the composition of the present sample, which included many former varsity student-athletes from endurance sports, and a smaller proportion from esthetic sports. These sport contexts may involve sport-specific ideals emphasizing leanness and weight control [66]. Within such disciplines, DE associated with drive for thinness may be more likely to emerge or be maintained because they align with salient performance and appearance standards [67]. Conversely, DE associated with drive for muscularity may be less salient or less valued because increased muscular mass can be perceived as detrimental in these sport types. In parallel, the control group may be more strongly exposed to broader sociocultural pressures that increasingly promote a “lean and muscular” ideal, potentially elevating muscularity-related concerns outside high-level sport contexts [65,68,69]. Finally, if DE associated with drive for thinness were normalized during athletes’ training years, those behaviors may persist after sport disengagement through habit formation and internalized performance norms, even when the competitive context is no longer present [7,10,13].

The association between perfectionism and DE (associated with drive for thinness and associated with drive for muscularity) tended to be stronger in the control group than in former student-athletes, contrary to the initial hypothesis. Rather than implying that varsity sport is inherently “protective,” this pattern may indicate that the way perfectionism translates into DE may differ by context. A context-based hypothesis is that perfectionism may be more directly expressed through appearance-focused goals in non-athlete settings, where sociocultural “lean and muscular” ideals provide a salient standard against which individuals evaluate themselves [65,68]. In contrast, it is possible that former student-athletes have learned to channel perfectionistic strivings toward performance-relevant domains (e.g., training quality, discipline, measurable outcomes) and to evaluate success using functional criteria rather than appearance criteria. If this is the case, perfectionism would remain present but be less tightly coupled with DE once sport norms and performance metrics structure self-evaluation. In fact, a recent study shows that a performance-oriented climate is associated with perfectionistic concerns in young athletes, suggesting that sport environments may shape how perfectionistic tendencies are expressed [70]. Specifically, athletes may learn to channel these tendencies toward performance-related goals, a pattern that may persist even after their transition out of sport. As the present data do not allow for testing this hypothesis, future research employing comparative designs across contexts is needed to further clarify the relationship between perfectionism and DE.

### 4.3. Orthorexia Symptoms

Orthorexia symptoms did not differentiate former student-athletes from controls, whereas perfectionism emerged as a significant predictor of orthorexia symptoms across participants. This suggests that, in the present sample, orthorexia symptoms were more closely tied to an individual vulnerability factor (perfectionism) than to group membership. This finding diverges from the initial hypothesis and from studies reporting higher orthorexia symptoms in athletes than in non-athlete controls [71,72]. A parsimonious explanation is methodological heterogeneity across studies, including differences in how orthorexia is operationalized (questionnaires and cut-offs), how “athlete” status is defined (current vs former athletes; competitive level; training volume), and how control groups are selected and matched [71,73,74]. Differences in sport type may be particularly relevant: Karaağaç et al., for instance, focused primarily on team sport athletes, whose performance demands, body ideals, and food-related norms can differ substantially from those of endurance or esthetic sports [71]. Variation in sport culture and normative eating practices may therefore contribute to inconsistent group differences across samples. These results support two priorities for future research. First, studies should compare student-athletes and former student-athletes across a broader range of sport categories and explicitly test sport type as a moderator. Second, given that former student-athletes displayed higher perfectionism than controls and that perfectionism predicted orthorexia symptoms, longitudinal designs should test an indirect pathway in which group differences in orthorexia symptoms, if they emerge over time, are mediated by perfectionism.

### 4.4. Other Considerations and Practical Implications

The absence of gender effects in main analyses is noteworthy. The variables examined in this study (particularly DE associated with drive for thinness and drive for muscularity) are documented as being gendered [11]. Several explanations may account for this discrepancy. First, statistical power and measurement precision may have been insufficient to detect gender differences. The marked imbalance between men and women likely reduced power for between-gender comparisons and may have widened confidence intervals, making true effects harder to detect even if they exist. Second, the apparent null effect may reflect confounding or suppression within the models. For example, if athlete status is strongly associated with the outcomes and unevenly distributed by gender, its inclusion can attenuate the unique contribution of gender. In this case, gender differences may not be absent but rather absorbed by variables more proximal to the behaviors studied. Third, it is plausible that gender contrasts are decreasing in these constructs because contemporary body ideals increasingly converge toward a “lean and muscular” standard across genders, potentially reducing historically observed differences in DE associated with drive for thinness and drive for muscularity [68,69].

In addition, only SOP was significantly associated with all three forms of DE. These findings are consistent with the transdiagnostic model [8], as they suggest that these DE may share common underlying psychological mechanisms. However, the observed differences between groups for certain forms of DE may point towards variability in how these difficulties manifest across contexts (e.g., depending on experience in high-level sport). As such, these results suggest that although DE may be underpinned by shared psychological processes, it is essential to consider its diverse forms and manifestations to achieve a more accurate and nuanced understanding of these difficulties across different populations and contexts.

Finally, these results point to some practical recommendations. Sports environments should be made aware of exercise addiction symptoms, and of the different possible forms of DE in athletes, during the sport career but also during sport retirement, in order to better identify, prevent, and intervene in these types of difficulties, which may become severe if left unaddressed [60]. Moreover, psychological interventions that reduce rigid perfectionistic standards and increase psychological flexibility may help buffer risk during sport retirement. This can be operationalized by targeting intolerance of mistakes, shifting self-evaluation away from body-based metrics, strengthening interoceptive and body awareness, and cultivating acceptance-based approaches to body image [75,76].

### 4.5. Limitations

Several limitations constrain interpretation of these findings. First, all constructs were assessed using self-report questionnaires, and the French version of the EMS has not yet undergone full psychometric validation. As such, findings related to DE associated with drive for muscularity should be interpreted with caution until further validation studies are conducted. Moreover, the instrument EDS-R mainly focuses on exercise dependence and may not fully capture the nuances associated with the compulsive dimension of exercise addiction [17]. Future research should incorporate clinician-administered interviews and multi-method assessment to strengthen validity. Second, the cross-sectional design precludes conclusions about temporal ordering and causality. The present data cannot determine whether symptoms preceded sport disengagement, emerged during the transition period, or persisted afterward. Longitudinal studies starting before retirement and extending into the post-sport period are needed to model symptom trajectories and to test prospective pathways (e.g., whether perfectionism predicts later symptom changes). Third, the geographic specificity of the sample (participants from Québec) and the sample composition limit generalizability and subgroup analyses. Indeed, given that Québec has a specific sport and educational system, this may limit the generalizability of the findings to other sport or educational contexts. Although adequate for the primary comparisons, the sample size and uneven distribution across sport categories prevented robust tests of sport-type differences, and the gender imbalance likely reduced power to detect moderation by gender. Larger, more balanced samples, including stronger male representation and sufficient numbers across sport types, would allow direct tests of sport type and gender as moderators of DE and exercise addiction symptoms, consistent with sport-specific risk variability [13]. Finally, although the group difference in the perfectionism–DE association was statistically significant, it accounted for only a modest proportion of variance in drive for thinness and drive for muscularity (2.8%). As such, these results should be interpreted conservatively. The data are exploratory and do not support a causal “protective effect” of athletic experience. Future studies should directly measure hypothesized mechanisms (e.g., internalization of appearance ideals, performance-oriented standards, identity transition factors, and sport norms) to explain why perfectionism may link to DE differently across former student-athletes and controls.

## 5. Conclusions

Taken together, these findings provide a more comprehensive understanding of exercise addiction symptoms and DE during sport retirement. Specifically, results indicate that former varsity student-athletes may experience a range of DE and symptoms of exercise addiction even after leaving high-level sport. They also support existing literature linking perfectionism to both DE and exercise addiction, while suggesting that the experience of being a varsity student-athlete may shape this relationship. These results underscore the importance of recognizing different forms of DE and exercise addiction symptoms early in the athletic career and of addressing perfectionism in prevention and intervention efforts, particularly during critical transitions such as sport retirement.

## Figures and Tables

**Table 3:** Regression and Moderation Models for DE Associated with Drive for Thinness (*n* = 150).

	Model 1: Linear Regression				Model 2: Moderation			
*R* ^2^	0.204 *				0.232 *			
Adjusted *R*^2^	0.159				0.183			
*F* (*df*1, *df*2)	3.688 (8, 141)				4.318 (9, 140)			
	*b*	*S.E*	*p*	*β*	*b*	*S.E*	*p*	*β*
Sex ^+^	0.138	0.281	0.623	0.049	0.203	0.282	0.473	0.072
Group ^++^	0.361	0.228	0.115	0.150	0.350	0.218	0.207	0.146
BFI_E	0.132	0.112	0.239	0.099	0.137	0.112	0.218	0.102
BFI_A	−0.126	0.236	0.594	−0.055	−0.086	0.233	0.712	−0.038
BFI_C	−0.007	0.169	0.969	−0.004	−0.040	0.174	0.821	−0.024
BFI_N	0.167	0.128	0.194	0.121	0.153	0.125	0.221	0.111
BFI_O	−0.082	0.152	0.593	−0.045	−0.057	0.151	0.705	−0.031
SOP	0.066	0.017	<0.001	0.388	0.031	0.017	0.001	0.340
SOP × Group	-	-	-	-	0.060	0.030	0.048	0.177

Note. ^+^ 0 = Male and 1 = Female. ^++^ 0 = former student-athletes and 1 = control group. SOP = Self-Oriented Perfectionism; BFI_E = Extraversion dimension of BFI; BFI_N = Neuroticism dimension of BFI; BFI_A = Agreeableness dimension of BFI; BFI_C = Conscientiousness dimension of BFI; BFI_O = Openness dimension of BFI. * *p* < 0.05.

**Table 4:** Regression and Moderation Models for DE Associated with Drive for Muscularity (*n* = 150).

	Model 1: Linear Regression				Model 2: Moderation			
*R* ^2^	0.203 *				0.230 *			
Adjusted *R*^2^	0.157				0.181			
*F* (*df*1, *df*2)	3.202 (8, 141)				4.413 (9, 140)			
	*b*	*S.E*	*p*	*β*	*b*	*S.E*	*p*	*β*
Sex ^+^	0.102	0.098	0.300	0.096	0.126	0.101	0.218	0.118
Group ^++^	0.197	0.081	0.016	0.216	0.193	0.078	0.015	0.213
BFI_E	0.017	0.039	0.665	0.033	0.018	0.037	0.620	0.036
BFI_A	−0.112	0.082	0.173	−0.130	−0.097	0.083	0.241	−0.113
BFI_C	−0.098	0.063	0.124	−0.157	−0.110	0.064	0.086	−0.177
BFI_N	−0.023	0.041	0.577	−0.044	−0.028	0.041	0.495	−0.054
BFI_O	−0.005	0.051	0.920	−0.007	−0.004	0.050	0.936	0.006
SOP ^++^	0.028	0.006	<0.001	0.433	0.015	0.006	0.108	0.386
SOP × Group	-	-	-	-	0.022	0.011	0.044	0.173

Note. ^+^ 0 = Male and 1 = Female. ^++^ 0 = former student-athletes and 1 = control group. SOP = Self-Oriented Perfectionism; BFI_E = Extraversion dimension of BFI; BFI_N = Neuroticism dimension of BFI; BFI_A = Agreeableness dimension of BFI; BFI_C = Conscientiousness dimension of BFI; BFI_O = Openness dimension of BFI. * *p* < 0.05.

**Table 5:** Regression Model for Orthorexia Symptoms (*n* = 150).

Model 1: Linear Regression				
*R* ^2^	0.200 *			
Adjusted *R*^2^	0.154			
*F* (*df*1, *df*2)	5.109 (8, 141)			
	*b*	*S.E*	*p*	*β*
Sex ^+^	−0.661	1.117	0.555	−0.053
Group ^++^	0.254	0.922	0.783	0.024
BFI_E	0.225	0.438	0.608	0.038
BFI_A	0.515	0.781	0.511	0.051
BFI_C	0.315	0.692	0.649	0.043
BFI_N	0.292	0.507	0.566	0.048
BFI_O	−0.063	0.718	0.931	−0.008
SOP	0.300	0.061	<0.001	0.404

Note. ^+^ 0 = Male and 1 = Female. ^++^ 0 = former student-athletes and 1 = control group. SOP = Self-Oriented Perfectionism; BFI_E = Extraversion dimension of BFI; BFI_N = Neuroticism dimension of BFI; BFI_A = Agreeableness dimension of BFI; BFI_C = Conscientiousness dimension of BFI; BFI_O = Openness dimension of BFI. * *p* < 0.05.

**Table 6:** Regression Model for Exercise Addiction Symptoms (*n* = 150).

Model 1: Linear Regression				
*R* ^2^	0.170 *			
Adjusted *R*^2^	0.123			
*F* (*df*1, *df*2)	4.592 (8, 141)			
	*b*	*S.E*	*p*	*β*
Sex ^+^	−3.342	3.884	0.391	−0.086
Group ^++^	−4.122	3.105	0.186	−0.125
BFI_E	−0.795	1.504	0.598	−0.043
BFI_A	−0.758	2.959	0.798	−0.024
BFI_C	1.609	2.288	0.483	0.071
BFI_N	0.893	1.757	0.612	0.047
BFI_O	2.378	2.216	0.285	0.095
SOP	0.691	0.210	0.001	0.299

Note. ^+^ 0 = Male and 1 = Female. ^++^ 0 = former student-athletes and 1 = control group. SOP = Self-Oriented Perfectionism; BFI_E = Extraversion dimension of BFI; BFI_N = Neuroticism dimension of BFI; BFI_A = Agreeableness dimension of BFI; BFI_C = Conscientiousness dimension of BFI; BFI_O = Openness dimension of BFI. * *p* < 0.05.

## Data Availability

The datasets analyzed during the current study are not publicly available due to participant consent conditions and research ethics requirements, but may be made available from the corresponding author, Juliette Maurin, at juliette.maurin@usherbrooke.ca, on reasonable request and subject to applicable research ethics requirements.

## Abbreviations

The following abbreviations are used in this manuscript:

ED	Eating disorders
DE	Disordered eating
EDE-Q	Eating Disorder Examination Questionnaire
EMS	Eating for Muscularity Scale
EFO	Échelle Française d’Othorexie
EDS-R	Exercise Dependence Scale-Revised
SOP	Self-oriented perfectionism
BFI	Big Five Inventory
BFI-E	Big Five Inventory-Extraversion
BFI-N	Big Five Inventory-Neuroticism
BFI-A	Big Five Inventory-Agreeableness
BFI-C	Big Five Inventory-Conscientiousness
BFI-O	Big Five Inventory-Openness

## Appendix A

**Table A1:** Correlations among study variables.

	1.	2.	3.	4.	5.	6.	7.	8.	9.	10.
1. EDE-Q	1									
2. EMS	0.698 *	1								
3. EFO-12	0.495 *	0.608 *	1							
4. EDS-R	0.188 *	0.395 *	0.483 *	1						
5. SOP	0.364 *	0.333 *	0.425 *	0.374 *	1					
6. BFI_E	0.035	−0.035	0.018	−0.058	−0.065	1				
7. BFI_N	0.251 *	0.190 *	0.102	0.058	0.202 *	−0.134	1			
8. BFI_A	−0.089	−0.173 *	0.014	−0.066	−0.078	0.233 *	−0.278 *	1		
9. BFI_C	0.079	−0.040	0.174 *	0.151	0.336 *	0.063	−0.230 *	0.306 *	1	
10. BFI_O	0.001	0.019	0.026	0.095	0.032	0.118	0.041	0.027	0.072	1

Note. EDE-Q = Eating Disorder Examination Questionnaire; EMS = Eating for Muscularity Scale; EFO-12 = Échelle Française d’Orthorexie; EDS-R = Exercise Dependance Scale Revised; SOP = Self-Oriented Perfectionism; BFI_E = Extraversion dimension of BFI; BFI_N = Neuroticism dimension of BFI; BFI_A = Agreeableness dimension of BFI; BFI_C = Conscientiousness dimension of BFI; BFI_O = Openness dimension of BFI. * *p* < 0.05.
